# Can Peer-based Interventions Improve Adolescent Sexual and Reproductive Health Outcomes? An Overview of Reviews

**DOI:** 10.1016/j.jadohealth.2023.05.035

**Published:** 2023-07-13

**Authors:** Amanda J. Mason-Jones, Marlon Freeman, Theo Lorenc, Tina Rawal, Shalini Bassi, Monika Arora

**Affiliations:** a Department of Health Sciences, University of York, York, United Kingdom; b Centre for Reviews and Dissemination, University of York, York, United Kingdom; c Public Health Foundation of India, Gurgaon (Haryana), India

**Keywords:** Peer-based approaches, Peer-education, Intervention, Adolescents, Sexual and reproductive health, Overview of reviews

## Abstract

**Purpose:**

An overview of reviews was conducted to summarize the evidence and synthesize the results from systematic reviews.

**Methods:**

The Cochrane and Preferred Reporting Items for Overviews of Reviews reporting guidelines were followed and the protocol was registered. Electronic and manual searches were conducted to identify systematic reviews, published between January 1990 and July 2022. Studies with outcomes relating to all areas of adolescent sexual and reproductive health (SRH) (changes in knowledge, attitudes, beliefs, skills, and practices) were considered. The ROBIS (Risk of Bias in Systematic Reviews) tool was used to assess quality.

**Results:**

A total 1849 articles were retrieved, and eight reviews met the inclusion criteria. Three of the eight reviews included meta-analyses. All three of these reviews demonstrated a significant improvement in HIV knowledge. One reported improved attitudes toward people living with HIV but none found any statistically significant effect on condom use or other SRH behaviors. The remaining five reviews included reports of positive individual study outcomes related to knowledge and attitudes and provided narrative syntheses with regard to recruitment, training, support, and participation of peers. Five of the eight reviews were judged to have a low risk of bias.

**Discussion:**

Our overview demonstrates that peer-based interventions can improve SRH knowledge and attitudes. Evidence of their effectiveness in promoting healthier SRH behaviors is less certain. Any future studies need to investigate which adolescent health outcomes peer-based programs could reasonably be expected to improve using robust methodologies. Additionally, peers need to be meaningfully engaged and acknowledged as experience-based experts.

Peer-based interventions are premised on the concept of lay community support for improving health and wellbeing. This approach has been co-opted by modern health care systems where resources or the capacity of health workers is limited, or because the target groups are marginalized in some way and can be theoretically reached more easily by peers. Where the purpose is to promote adolescent sexual and reproductive health (SRH), the shared affinity, experience, and understanding of the challenges involved in being an adolescent could be powerfully garnered to create positive influence to improve health and wellbeing outcomes [1–3]. Additionally, it has been suggested that peer-based programs may benefit peer leaders themselves by increasing their knowledge and inspiring them to be agents of change in their communities [4].

Many individual studies have explored peer-based interventions for improving adolescent SRH outcomes in a number of countries including South Africa [5], Cambodia [6], and England [7] although with mixed results.

This may be because the theoretical basis for peer-based approaches has not always been clear and, as a result, the logic model remains incomplete [8]. This has led some authors to question it as a suitable approach when it comes to promoting adolescent SRH [9,10]. However the popularity of the peer-based approach with governments [11–13] has meant that it has been implemented and evaluated globally. However, to our knowledge, there has never been an overview of systematic reviews conducted before. The overview of systematic reviews can provide insight that is missing from individual reviews to help provide decision makers and researchers with a clearer understanding of the topic [14]. The aim of this overview was to explore the effectiveness of peer-based interventions for improving adolescent SRH as evident from review level evidence.

## Methods

The overview followed the methods suggested by the Cochrane collaboration [15,16] and used the emerging guidance for reporting [17,18]. The protocol was registered with the International Prospective Register of Systematic Reviews (PROSPERO CRD42017076290).

### Inclusion Criteria

#### Study designs

Systematic reviews of effectiveness of peer-based interventions were considered that were published in English. As interchangeable terms are often used a study was considered to be a systematic review if it included a detailed search strategy and inclusion criteria for the studies reviewed [19]. Qualitative designs were excluded.

#### Population

The World Health Organization definition of ‘adolescent’ was used with the age range for participants in the reviews between 10–19 years of age [20]. We included studies that had a wider range of ages as long as the systematic review had some focus on adolescents aged 10–19 years regardless of overall age range. Reviews from any region of the world were included.

#### Intervention

The interventions of interest were peer-based (described variously as peer education, peer-counseling, peer-led, peer-driven, peer-tutored, peer-facilitated, peer-assisted) and were focused on improving SRH outcomes. If the systematic review included other types of intervention, then at least three included studies must have included peer-based interventions focused on adolescent SRH. All SRH topics were included such as information or training about pregnancy and contraception, sexually transmitted infections (STIs) including HIV/AIDS, and other aspects of relationships, and SRH. The peer-based intervention could include any size of group or one-on-one interactions and could be implemented in any kind of setting such as schools, and/or communities. They could be either curriculum or noncurriculum based, and programprograms of any duration were included.

#### Outcomes

Systematic reviews that measured outcomes including change in adolescents’ knowledge, attitudes, beliefs, skills, and practices in relation to SRH were included.

### Databases

EMBASE, Medline, ASSIA, and CINAHL were searched between January 1990 – July 2022. Ovid database host was used to search EMBASE and Medline, EBSCO database hosted CINAHL, and ProQuest database hosted ASSIA. Reference lists were also searched for any additional reviews.

### Search strategy

The search strategy was developed with a combination of search terms and Medical Subject Heading phrases for the following keywords: ‘peer’, ‘adolescent’, ‘sexual and reproductive health,’ and ‘systematic review’. In addition to the advanced filter and the standard Medical Subject Heading vocabulary system we were also able to gather a comprehensive list of synonyms for our search [21]. The search strategy was then adapted using customized truncations and field codes for each database to optimize the search syntax [22]. Table 1 shows the search strategy used for CINAHL.

### Data management

EndNote reference management software was used to organize and deduplicate references.

**Figure 1:**
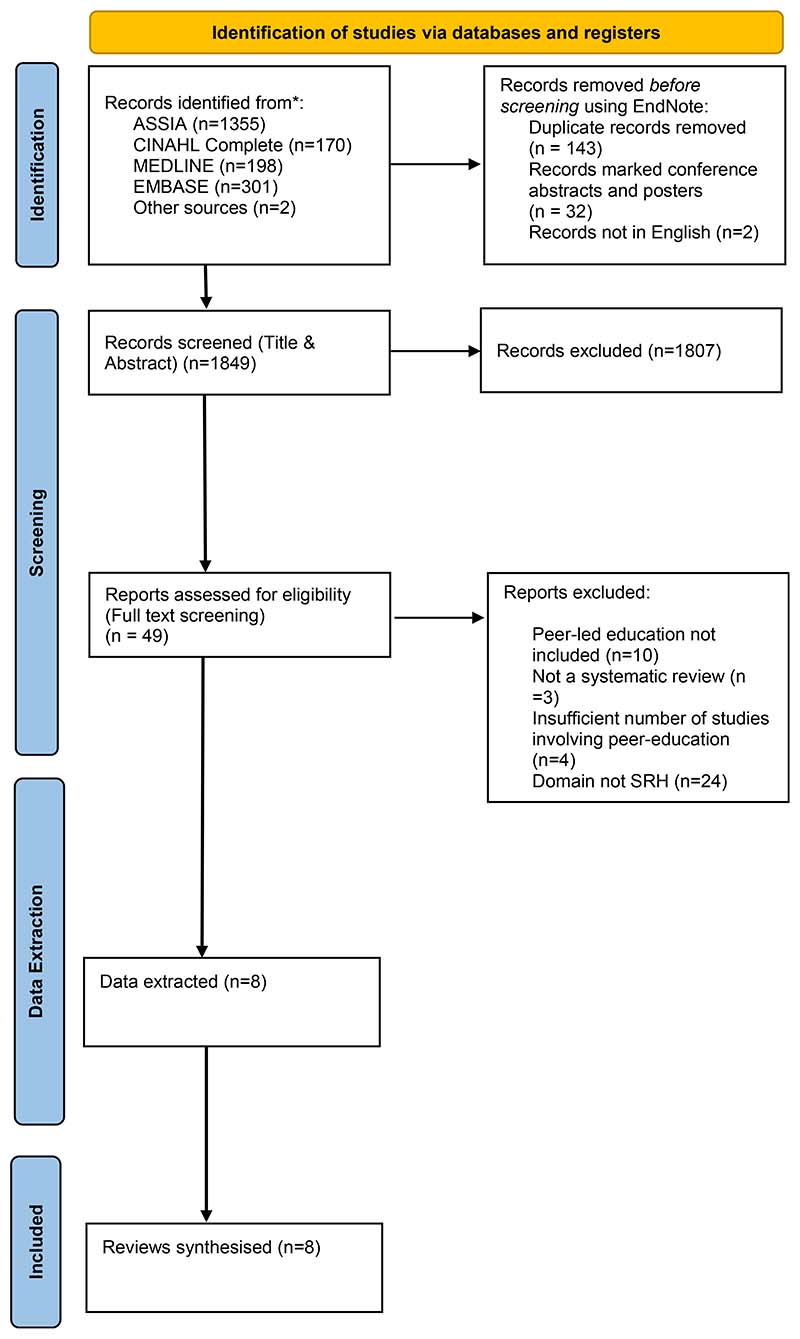
PRISMA flow diagram. From: Page MJ, McKenzie JE, Bossuyt PM, Boutron I, Hoffmann TC, Mulrow CD, et al. The PRISMA 2020 statement: an updated guideline for reporting systematic reviews. BMJ 2021;372:n71. doi: 10.1136/bmj.n71. For more information, visit: http://www.prisma-statement.org/

Independent screening of titles and abstracts was conducted by at least two independent reviewers (MF/TL, or MF/AMJ). Full-text screening then took place with data extraction completed by one reviewer (MF or AMJ) and a 15% sample reviewed by the second reviewer (AMJ or TR) to ensure consistency. During full-text screening, the reasons for exclusion were recorded and included in the Preferred Reporting Items for Systematic Reviews and Meta-Analyses (PRISMA) flow diagram (Figure 1).

### Data extraction

A bespoke form was developed to extract data from the reviews. Table 2 summarizes the information extracted from the included reviews. Additionally, all included individual studies were recorded, and overlaps between reviews were charted. The data extraction was piloted by two independent reviewers (AMJ, TR) using three included reviews to ensure consistency.

### Risk of bias

The ROBIS (Risk of Bias in Systematic Reviews) tool was used to assess the risk of bias [23] (Table 3). Three phases and multiple domains that use signaling questions were used to assess and highlight any concerns about any potential bias in each of the included reviews. This assessment was recorded on the data extraction form (MF or AMJ), and the judgment was checked by a second reviewer (AMJ or TR).

### Data synthesis

Meta-analyses were reported and a narrative approach to synthesis was used to combine the evidence from the included reviews. Major themes were extracted to explore the similarities, differences, and relationships between the reviews as suggested by other authors such as the synthesis without meta-analysis (SWiM) [24], guidance on the conduct of narrative syntheses [25], and the Preferred Reporting Items for Overviews of Reviews guidelines [26].

## Results

The search resulted in 1849 articles following deduplication. After the title and abstracts were screened, 49 full text articles and reports were reviewed, of which, eight were included in the analysis (See Figure 1).

The included reviews were published between 2006 and 2020 and comprised a total of 61 individual studies. Reviews included between 3 [27] and 16 [28] studies from a wide range of countries and all regions of the world. One review was specifically focused on India [29], one on the northern European region [30], one on sub-Saharan Africa [27], two focused on a selection of low-income and middle-income countries [31,32], one on ‘more developed countries’ [33], and two on all regions [28,34]. There was some overlap in the studies included in the reviews. For example, Agha 2004 [35] was included in four reviews [27,31,32,34], Brieger 2001 [36] in 4 [27,28,31,34], Borgia 2005 [37] in 3 [30,33,34], Speizer 2001 in 3 [28,32,34], Stephenson 2008 in 3 [30,32,33], Kinsler 2004 [38] in 2 [31,34], Ozcebe and Akin 2002 [39] in 2 [28,34], Mellanby 2001 [40] in 2 [30,34], and Merati 1997 [41] in 2 [28,32]. However, no discrepancies were found between the reporting of these studies in the reviews.

### Study designs and outcome measures

All of the reviews included quantitative studies that used a randomized controlled trial design (both individual and cluster randomized trials), quasi-experimental studies or controlled before and after studies. Outcome measures were largely self-reported although three of the reviews [30,32,33] included a study that used routine live births and abortion data [7].

### Types of synthesis and results

Three of the eight reviews conducted meta-analyses of trials using pooled estimates with odds ratios (OR) and 95% confidence intervals of the estimates reported or Hedges’ g and confidence intervals comparing the effect size of differences between groups [32–34].

Medley and colleagues [32] found statistically significant odds of improvement in HIV knowledge (OR 2.25, 95% CI 1.62, 3.92) while Sun and colleagues’ meta-analysis of seven studies [33] found that Hedges’ g of HIV knowledge change was 0.84 (95% CI 0.43, 1.25) and represented a large effect size (>0.8). For condom use, all reviews that included a meta-analysis found no statistically significant effect (Medley, (OR: 1.12; 95% CI 0.85, 1.48) [32], Sun, (OR 1.01, CI 0.88, 1.15) [33], and Kim, (OR 1.06 95% CI 0.92–1.21) [34]. Sun and colleagues [33] also found that peer-based programs improved young people’s attitudes toward people living with HIV and AIDS (Hedges’ g 0.49, 95% CI 0.19, 0.80). The heterogeneity of included studies was explored in two reviews [32,34]. Both found that there was substantial heterogeneity across studies, although where studies were analyzed by subgroup, particularly in terms of the selection and recruitment process of peers, they did suggest homogeneity. Nevertheless the authors cautioned that this could be a statistical artifact [34]. These reviews therefore also included a narrative synthesis to supplement their analyses.

The remaining five reviews reported statistically significant improvements from the individual included studies. This included improved knowledge of HIV and other STIs [27–29], knowledge of puberty, menstrual hygiene, contraception, complications in pregnancy and childbirth, reproductive tract infections and the existence of services [29], improved attitudes toward people living with HIV and consistent condom use [29,30], better communication about condom use, increased condom use, modern contraception, and condom self-efficacy [27–29].

Two of the reviews reported an *increase* in the initiation of sexual activity in the peer-based intervention group in the previous three months [30,31]. However, the authors of the individual paper, (reported in both reviews), did not appear to adjust their analysis for baseline imbalance of sexual activity between the intervention and control groups. Additionally, the follow up survey for this study was not applied to the same sample as the baseline [36].

### Peer recruitment, training and support

A range of methods were used to recruit peers including volunteering, the most common method reported, [27,28,32–34] nomination or recruitment by others including peers, [28,32], being chosen by teachers [30] or less commonly through literacy-based oral and written exams [34]. One review did not mention the method of recruitment of peers in the included studies [29]. It was noted that recruitment that involved volunteering resulted in more female than male recruits [34].

Training time, support and supervision for peer leaders differed across reviews. The shortest training time mentioned was one hour on one day [33,42] to around 60 hours over a few months [30]. However it was noted that the cascade model (peers training other peers) tended to be more challenging to implement and those programs that ran for longer (some for up to 4 years) benefited from refresher training to maintain enthusiasm and to ensure program fidelity [28]. One review did not mention any kind of supervision [31] whilst another noted that a lack of supervision did not necessarily result in poorer outcomes [28]. However, the individual study referred to was based in Russia and was an HIV prevention program focused on people who used intravenous drugs. It used a cascading network model, where peers were offered modest coupon-based incentives to recruit, educate, and follow up with their peers [43]. One of the reviews also focused on the participation of peers based on Hart’s ‘ladder of participation model’ [44] but found only two of the 15 included studies provided peer leaders with ‘high responsibility’ [33].

### Topics, activities, and delivery methods

Topics and activities were varied and included the effectiveness and impact of SRH programs generally [34] or in specific country regions (India [29], Europe [30], sub-Saharan Africa [27], or ‘developing’ countries [45]). They covered HIV risk, contraception, and condom use worldwide [28], HIV and associated risks in ‘developing’ countries [32], HIV, pregnancy, and sexual health promotion [30], HIV and STI knowledge, contraception and sexual violence in ‘more developed’ countries [33].

A range of methods were used by peer leaders including lectures and group-based sessions [27,29–33], role play [27,28,30], one-on-one counseling sessions [27,28], community events [27], information kiosks and anonymous question boxes [30], online and Facebook pages [33], and referrals onto more specialist services [28].

Some reviews reported that information on what were termed ‘life skills’ were also included [28,33]. These included learning skills that challenged social norms, debating, and other skills considered useful for improving SRH and potentially transferrable to other parts of the adolescent’s life [28,31]. Some studies demonstrated sensitivity in dealing with adolescent SRH. For example, by using anonymous question boxes where young people could post their questions so that conversations could be facilitated and privacy maintained [30]. Some peer interventions were delivered alongside other interventions including multi-component programs that included targeting teachers or capacity building for health workers [29].

### Quality assessment of the reviews

The quality of the included reviews was assessed using the ROBIS tool. Five reviews, (Kim and Free [34], Medley et al. [32], Maticka-Tyndale and Barnett, [28], Sun et al., [33] and Tolli, et al. [30]), had “low Concern” across all domains and were consequently judged to be at low risk of bias. These reviews included clearly defined and unambiguous inclusion criteria, a detailed search strategy and additional methods for finding relevant studies. The tools used for data extraction and appraisal of the studies and evaluations of the decisions taken by the researchers regarding the synthesis methodology were robust. Two reviews included one domain that was scored “unclear concern”; Kalembo [27] did not provide sufficient detail with regard to data collection and appraisal, whilst Kirby, [31], included insufficient detail with regard to synthesis and specifically how heterogeneity between studies had been addressed. Siddiqui and colleagues [29] provided insufficient information in both of these domains. So, whilst the included reviews were largely of sufficient quality there were some minor gaps in reporting.

## Discussion

This overview of systematic reviews aimed to synthesize what is known about the effectiveness of peer-based interventions for improving adolescent SRH from review level evidence. Peer-based approaches were successfully utilized to increase knowledge and used a wide range of methods of intervention delivery. Three of the reviews were able to conduct meta-analyses which showed that peer-based interventions could increase knowledge and create shifts in attitudes, although this did not necessarily translate into changing behaviors.

Differences between peer-based interventions included recruitment method. The most commonly reported method of peer recruitment was volunteering rather than being nominated by their own peers. The type of delivery methods varied as did the measurement of outcomes, duration of the intervention, and period of follow up. Additionally, the involvement, support, and supervision of the peer leaders were reported to impact the implementation and outcomes of the included programs. The process of supervision, training and support of peer leaders was often sub-optimal.

The overview benefited from a prespecified protocol registered with PROSPERO and the use of the ROBIS tool to assess the risk of bias of the included systematic reviews. Assessment using the ROBIS tool showed low concern of bias across all the domains in five out of the eight included reviews [28,30,32–34]. The remaining three studies had low concern of bias in at least two of the four domains [27,29,31].

Although all of the reviews were limited to English, the reviews themselves also included individual studies that were published in other languages. A very wide range of individual studies were included from all regions of the world and there was limited overlap of studies between reviews.

A range of SRH knowledge, attitudes and behaviors were the focus of this overview although, perhaps because of the age of many of the included studies, HIV prevention tended to be the main focus. As knowledge of HIV has become ubiquitous, perhaps using peer-based approaches where new knowledge is needed in other areas of SRH will be fruitful. Menstruation, endometriosis, abortion, and testicular cancer along with other SRH issues, were largely absent as were issues around intimate partner violence, sexual violence more generally, and mental health. More exploration is therefore needed to find out which topics, knowledge and issues the peer-based method of delivery might be most appropriate for. Menstruation was the subject of another review that included one study out of scope for this overview [46] but suggested that peer-based approaches can work in this area too.

The training, guidance, and follow up available to peer leaders also needs to be explored further. Kalembo and colleagues [27] noted that many programs in sub-Saharan Africa involved initial training but no refresher training or ongoing supervision, while peers involved in studies conducted in the USA received continued supervision during the intervention programs. Tolli’s review [30], focused on European countries, suggested that supervision and training was sub-optimal in this region too and did not follow the European guidance [47]. Nevertheless, one study provided supervision twice a month for its peer-leaders [48]. However, it was not possible to determine exactly how supervision and training influenced outcomes in this study.

There were a range of methods used which included group and one-to-one peer counseling. These may mimic some traditional pedagogic and health education approaches where a teacher, trainer, or other cadre of personnel facilitates an educative lesson and then offers one-to-one input. The use of peer-leaders may have the potential to create better engagement with content that is considered to be difficult to talk about [27,32]. Another method used was role play facilitated by the peer-leaders, which included practicing communication techniques that adolescents could use to avoid unwanted sex and to negotiate the use of condoms [28,31]. The use of such methods ignores the complex impact of inequity, violence, and power in all forms that impact sexual relationships. Partnership and support [49] and a rights focused intersectional framework [50] can incorporate the structural and ideological barriers that limit young people’s sexual and reproductive freedoms.

The range of justifications used by governments and researchers for the introduction of peer-led programs for adolescents (equal power relations, ability to talk about taboo topics, the potential for wider dissemination) are well aligned for ensuring sexual and reproductive rights [51]. However, program design is rarely informed by clear theoretical frameworks which may explain, in part, the disappointing results in terms of changing SRH behaviors. One review that explored the inclusion of a theoretical framework found that only eight of the 24 individual studies included one [28]. The search for a sound and consistent theoretical framework for peer-based approaches has been ongoing for over two decades [52]. The artificial reconstruction of social processes remains problematic, particularly for sexual and reproductive health [53] and many programs continue to focus on individual and proximate, rather than distal drivers of SRH behavior [54] and adolescent wellbeing.

Recent research exploring the role of social status in adolescent social network processes and health behavior has suggested that understanding and using these processes in intervention design is important yet often missing [55]. It has recently been employed in a pilot feasibility study that aims to “capitalize on mechanisms of social influence” [56]. Linked to this idea, a recent 10 year follow up mapping and qualitative evaluation of the ‘Toward Economic and Sexual/Reproductive Health Outcomes for Adolescent Girls’ program that used peer-based solidarity groups in Ethiopia to improve the wellbeing of married adolescent girls [57], found that this was effective and that increasing awareness of and access to services was crucial to this success.

Benefits for peers themselves are something that was rarely discussed in the reviews although one review reported that peer leaders reported increased social capital [28]. Benefits for peer-leaders have been explored in a South African government peer-based program [58] and found that interventions that remain individualized and do not recognize the socio-political and economic factors that impact young people’s lives may not be effective. The lack of material compensation for peer leaders can worsen the attrition to programs already beset by issues such as peer leaders ‘aging out’ of programs or faced with competing commitments such as family, work, or education that demands more of their time and focus [28].

Peer-based approaches to improve SRH have been applied globally but Harden and colleagues’ guidance [59] seems to have remained largely unheeded for SRH programs included in this overview. Therefore, as Siddiqui and colleagues argue, it may not be time to abandon this approach completely [29], particularly when it can be successfully used to effectively improve knowledge and change attitudes. For example, peer leaders from a national adolescent health program have been deployed during the COVID-19 pandemic [60] with admirable commitment and enthusiasm. What is needed going forward is a clear framework focused on reproductive justice [50], the full involvement of young people in the design of programs, acknowledgment of them as experience-based experts, and the provision of material compensation for their labor in any future high-quality evaluations.

## Figures and Tables

**Table 1:** Search strategy for CINAHL

S9	S8 and S7 and S6 and S5
S8	peer led OR peer leader* OR peer educat* OR peer tutor* OR peer facilitator* OR peer advisor* OR peer worker OR peer base* OR peer support* OR peer group* OR peer mentor* OR peer counsel* OR peer observ* OR peer outreach* OR peer moderat* OR peer observer OR peer outreach* OR peer mediat* OR peer deliver* OR peer model* OR peer facilitate* OR peer to peer
S7	adolescen* OR teen* OR young adult OR student* OR juvenile* OR youth* OR underage* OR Adolescent OR young people/person [MeSh]
S6	school or college [tw]
S5	S1 OR S2 AND S3 AND S4
S4	(literature OR articles OR publications OR publication OR bibliography OR bibliographies OR published OR pooled data OR unpublished OR citation OR citations OR database OR internet OR textbooks OR references OR scales OR papers OR datasets OR trials OR meta-analy* OR clinical) AND (studies OR treatment outcome OR treatment outcome OR pmcbook)
S3	(systematic OR systematically OR critical OR study selection OR predetermined OR inclusion) AND (criteri* OR exclusion criteri* OR main outcome measures OR standard of care OR standards of care)
S2	(systematic OR systematically OR critical OR study selection OR predetermined OR inclusion) AND (criteri* OR exclusion criteri* OR main outcome measures OR standard of care OR standards of care)
S1	(systematic review OR meta-analysis OR meta-analysis OR systematic literature review OR this systematic review OR pooling project OR systematic review) AND (meta synthesis OR meta-analy* OR integrative review OR integrative research review OR rapid review OR umbrella review OR consensus development conference OR practice guideline OR drug class reviews OR cochrane database syst rev OR acp journal club OR health technol assess OR clinical guideline) AND (management OR evidence based OR evidence-based medicine OR best practice* OR evidence synthesis) AND (review OR diseases category OR behavior and behavior mechanisms OR therapeutics OR evaluation studies OR validation studies OR guideline OR pmcbook)

**Table 2:** Summary of data extracted from the reviews

Review details	Authors Publication reference Citation Study registry
Review Characteristics	Number of included studies Type of included studies Date of the search for review Review objectives Intervention of interest Location of included studies
Summary information about adolescents in included studies	Age Gender Social demographics Training for peer-leaders Recruitment of participants Incentives provided
Summary information about the peer-led education intervention	Type of facilitation (group/one-on-one/ etc.) Topics covered Frequency and duration of intervention Curriculum or non-curriculum Details of supervision of intervention Any other information provided by study
Effects summary	Study findings Analytical results if meta-analysis or any statistical analysis Type of synthesis
Summary of quality of included studies	Describe details if any of type of quality assessment used for the included studies done by the review Risk of bias of the review
Limitations	Report any funding or conflict of interest

**Table 3:** Results of ROBIS quality evaluation

First author and year	Phase 2						Phase 3
	Concerns regarding specification of study eligibility criteria	Concerns regarding methods used to identify and/or select studies	Concerns regarding methods used to collect data and appraise studies	Concerns regarding methods used to synthesize results			Judging risk of bias
Kalembo, 2013	Low Concern	Low Concern	Unclear Concern	Low Concern			Unclear Risk of Bias
Kim, 2008	Low Concern	Low Concern	Low Concern	Low Concern			Low Risk of Bias
Kirby, 2006	Low Concern	Low Concern	Low Concern	Unclear Concern			Unclear Risk of Bias
Maticka-Tyndale, 2010	Low Concern	Low Concern	Low Concern	Low Concern			Low Risk of Bias
Medley, 2009	Low Concern	Low Concern	Low Concern	Low Concern			Low Risk of Bias
Siddiqui, 2020	Low Concern	Low concern	Unclear Concern	Unclear concern			Unclear Risk of Bias
Sun, 2018	Low Concern	Low concern	Low Concern	Low concern			Low Risk of Bias
Tolli, 2012	Low Concern	Low Concern	Low Concern	Low Concern			Low Risk of Bias
